# Gyro-Sensor-Based Vibration Control for Dynamic Humanoid-Robot Walking on Inclined Surfaces

**DOI:** 10.3390/s20247139

**Published:** 2020-12-12

**Authors:** Sunandan Dutta, Mitiko Miura-Mattausch, Yoshihiro Ochi, Naoto Yorino, Hans Jürgen Mattausch

**Affiliations:** 1 Graduate School of Engineering, Hiroshima University, Higashihiroshima, Hiroshima 739-8527, Japan; yorino@hiroshima-u.ac.jp; 2 HiSIM Research Center, Hiroshima University, Higashihiroshima, Hiroshima 739-8530, Japan; mmm@hiroshima-u.ac.jp (M.M.-M.); yoshihiro-ochi@hiroshima-u.ac.jp (Y.O.); hjm@hiroshima-u.ac.jp (H.J.M.)

**Keywords:** humanoid robot, gait analysis, gyro sensor, fourier analysis, inverted pendulum model, vibration

## Abstract

An efficient motor-control system for stable walking of the lightweight humanoid robot KONDO KHR-3HV on inclined surfaces is investigated. The motor-control system is based on the angular velocity of the pitch motion of the robot, which is detected by a gyro sensor attached to the robot torso and referred to as the angular-pitch velocity. The robot gait is analyzed for different downslopes with and without the motor-feedback control. A novel method of frequency-domain analysis of the angular-pitch velocity is proposed for explaining the reasons behind the instabilities of dynamic humanoid-robot walking on inclined surfaces. The results show, that a nonlinear nature of the motor torque, due to a force induced by the slope, gives rise to harmonics of the fundamental walking frequency of 1.73 Hz. These harmonics are the origin of the unstable robot walking. Additionally, the feedback-gain parameters *K*_A_ and *K*_H_ affect the amplitudes of the harmonics, which give rise to vibrations at a higher surface inclination. Increased surface friction allows a reduction of the feedback gain, which reduces this specific contribution to the harmonics and thus stabilizes the robot. To improve the walking stability on inclined surfaces, it is found that the damped natural frequency of the motor-control system must be kept lower than the fundamental walking frequency.

## 1. Introduction

**Figure 1:**
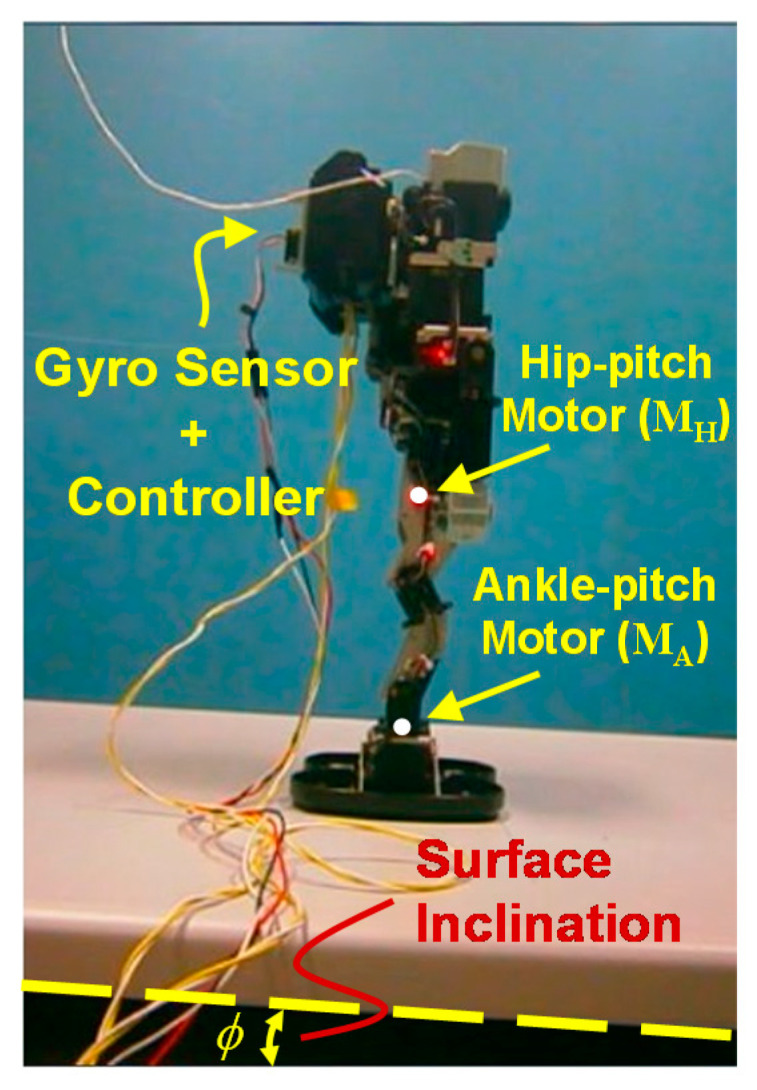
KONDO KHR-3HV humanoid robot integrated with a gyro sensor for balancing on an inclined surface with the inclination angle *φ*.

**Figure 2:**
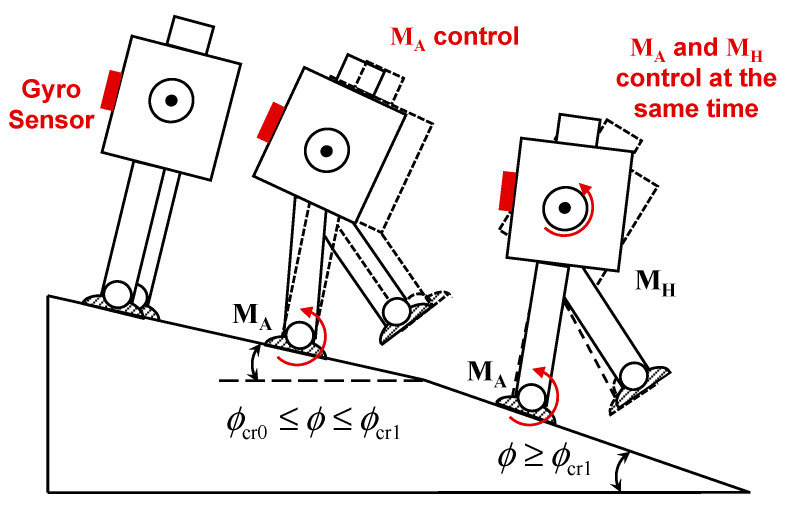
The gyro sensor measured angular-pitch velocity of the robot body is used to control the ankle-pitch motor (M_A_) above a critical inclination *φ*_cr0_ and is then used additionally to control the hip-pitch motor (M_H_) above a larger critical inclination *φ*_cr1_, in order to balance the robot walking.

Robots are important means to sustain as well as to develop a comfortable society [1,2,3,4,5,6]. The intensive utilization of robots has already been realized to reduce the necessity of human efforts for different purposes such as agricultural automation [1,2], inspection and maintenance [3,4], or rescue operations [5,6,7]. Presently, humanoid robots are also being developed to support human beings in various other tasks [6,8,9,10]. In an environment, where human beings act and dwell, it is especially practical and useful to develop robots of the humanoid type for supporting these human beings. Recent robotics challenges [11] have showcased humanoid robots for various tasks, which are difficult to be performed by human beings, e.g., as underground surveying, operations in mineralogical sites, or inspection of nuclear power plants. There has been large progress in implementing intelligent humanoid robots in various adverse and hostile environments [12,13,14]. Several model-based control techniques, such as those applying the Inverted Pendulum Model (IPM) [15] and its variations [16,17] or optimization-based trajectory planning and control [13,14] have been proposed in order to achieve a stable robot walking. One of the very fundamental and widely used control techniques is based on the Zero Moment Point (ZMP) [18,19], where the projection of the Center of Mass (COM) of the robot has to be kept always within the support polygon of the robot [19]. Simultaneously, environment modeling is also undertaken to enable the robot to switch between various control strategies [20,21,22,23,24]. Most of these control strategies use vision-based data to classify [20] or model and map [21,22] the environment for an improved control of the robot motion [23,24]. However, the development of a robust and environment-adaptive humanoid robot is not yet practically implemented in the society. A major problem of these model-based control techniques is the still insufficient consideration of the interaction between robot and environment. While many models include the interaction with a given set of environments, they fail to efficiently control the robot for other environments. It is very difficult to design a control system, which will stabilize a humanoid robot for all types of environments. Therefore, it is important to analyze the dynamics of the robot for each given environment. Terrain-blind robots, in which no visual data is available from the environment, i.e., no camera is used to survey the environment, are extensively studied in literature [25,26,27]. This terrain blindness constitutes the worst-case situation, since the robot must be solely controlled on the basis of physical interactions with the environment. Our focus is given on a motor-control scheme for balancing of the robot walking on an inclined surface, as shown in Figure 1. We have experimentally studied our robot-balancing scheme for different downslope inclinations (see Figure 2), using the lightweight humanoid robot KONDO KHR-3HV [28].

The most important feature of an ideal human-walking pattern or “gait pattern” is observed in its periodicity [29], and this periodic gait pattern is defined from the physiological point of view. Various experiments have been carried out by prosthetists, to assess the human gait-pattern [29,30,31,32,33,34,35,36,37,38,39] and include an analysis of basic parameters, such as the velocity or number of steps per unit time [30]. The joint angle of the robot leg and the ground-reaction force are most often studied among all the different parameters, to characterize the periodicity of the human gait [29,30,31,32,33]. For the characterization purpose, a frequency analysis of these measurable quantities is often carried out using the Fourier series expansion [31,32,33]. However, such a Fourier analysis was in most cases limited to the study of human patients, to determine the change in their walking dynamics due to various physical problems and diseases [36]. With the development of an Inertial Measurement Unit (IMU), based on a Micro Electro-Mechanical System (MEMS), the studies of orientation and force at each of the joint angles for human beings or humanoid robots have become more advanced [38,39,40].

In this paper, we have investigated the angular-pitch velocity, i.e., the angular velocity of the robot’s pitch motion in the sagittal plane, while the robot walks on the inclined surface. The angular-pitch velocity, considered in this paper, is therefore a single-dimension vector quantity. Clockwise and counter-clockwise rotation are denoted by positive and negative values of the velocity, respectively. We have measured the angular-pitch velocity with a single-axis analog gyro sensor KRG-4 [41] (see Figure 1), which is attached to the robot-torso. The sensitivity of the gyro sensor is 0.67 mV∙s/degree. The angular-pitch velocity of the robot is measured while walking on surfaces with different inclinations. Intensive investigations of the robot-walking characteristics, based on the angular-pitch velocity, have been undertaken.

To analyze the periodicity of any physical signal, the frequency-domain analysis is considered to be a fundamental approach. In the humanoid-robotics field, the Fourier series expansion is applied to generate the gait pattern for the robot’s basic walking motion, which can be modeled as a superposition of sinusoidal joint-angle variations [42,43]. Kajita et al. [44] used the frequency-domain analysis to suppress vertical vibrations in the HRP-4C robot. In [45], a frequency-domain classification of surfaces, based on the robot vibrations, is reported but not yet applied to the humanoid-robot control. Liu et al. [46] designed a vibration-reduction controller for elastic joints, implemented in a humanoid manipulator. The terminologies of resonant and anti-resonant frequencies with respect to the compliance modeling in motors were discussed in detail, highlighting the frequency-domain analysis of the vibrations. However, the control system was only applied to static manipulators, while the vibration effect on humanoid-robot dynamics was not reported. Very few works on the stability of humanoid robots, which exploit the Fourier analysis of the gait-data, have been reported. Kim et al. [47] reported vibration responses during leg lifting of a humanoid robot in the swinging phase, but did not adequately discuss the overall effect on the gait data, e.g., on the COM position or on the angular velocity components of the robot’s COM.

**Figure 3:**
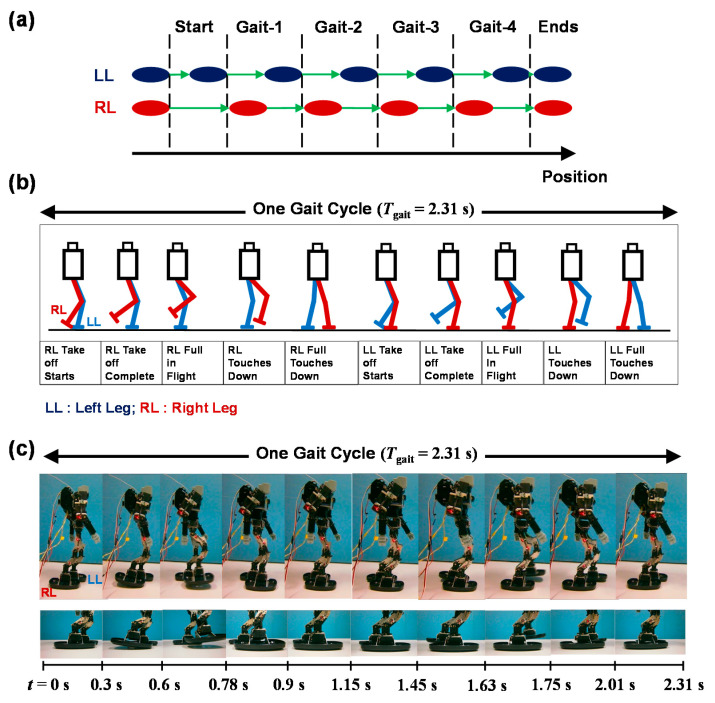
(**a**) Walking-motion scheme for the robot experiments, illustrating the gait-cycle repetition of the robot, with one half of a gait cycle at the beginning, then four complete gait cycles and finally one half of a gait cycle at the end. (**b**) One gait cycle is zoomed to show the gait pattern of the robot KONDO KHR-3HV. *T*_gait_: Period of a gait cycle; *T*_walk_: Total time for which the robot has walked. (**c**) Photos showing the gait pattern implemented in one gait cycle of the robot with the zoomed foot-placements patterns at the bottom.

Consequently, the purpose of our reported investigation is to analyze the balancing mechanism of a humanoid robot on inclined surfaces, to achieve stable walking with the help of motor-feedback control and Fourier analysis of the robot’s angular-pitch velocity, which is measured for one gait-cycle during the experiments, as shown in Figure 3. The angular-pitch velocity of the robot is measured while walking on different surface inclinations and analyzed in the frequency domain, where the origin of the observed periodicity disturbances of the robot gait is investigated. The novel contributions of the reported work include:

- Proposal of a gyro-sensor-based feedback-control system to control the ankle-pitch and hip-pitch motors of the robot for stable walking on inclined surfaces, as indicated in Figure 1. The control system enables the robot to walk stably on a downslope surface of inclination up to 10.2°. The feedback controller is easy to implement in a commercial, low-cost, mass-produced, and open-source hardware platform that can be integrated easily into a light-weight humanoid robot (see Section 2).
- The ankle-pitch and hip-pitch motors play a significant role in posture stabilization [15,19]. Therefore, initially for smaller surface inclinations, the ankle-pitch motor is controlled and for larger surface inclinations both ankle-pitch and hip-pitch motors are controlled. This reduces the computational complexity compared to the inverse-kinematics-based approaches in the conventional ZMP-based control [18,19,48] (see Section 2).
- The angular-pitch velocity of the robot is considered to be a characteristic of the robot-gait, is measured by the gyro sensor for the walking robot and is analyzed in the frequency domain. A novel use of the Fourier analysis for the angular-pitch velocity is proposed to determine the cause of postural instability on inclined surfaces. Moreover, the effect of the feedback gain on the robot gait on inclined surfaces is analyzed (see Section 2 and Section 3).
- Experimental observation, which increased the friction between robot feet and inclined surface reduces the robot vibrations at larger surface inclinations. The results of robot-walking experiments with increased friction represents an optimization approach for the feedback gain to reduce vibrations in the robot at increased surface inclinations (see Section 4).
- The IPM, used to model the robot walking, is extended for inclined surfaces. An additional gyro-sensor-based feedback loop is included in the model to explain the Fourier response of the angular-pitch velocity. Further, the IPM is extended to include the nonlinearity, induced by the surface inclination, to explain the harmonics observed in the Fourier transform of the angular-pitch velocity (see Section 5).

The section structure of this paper is described as follows. Section 2 describes the experimental setup and results of the walking experiments, carried out with the humanoid robot on an inclined surface with a low surface friction. In Section 3, the angular-pitch velocities of the robot, obtained from the experiments (discussed in Section 2), are analyzed in the frequency domain. Section 4 describes the experimental results for the robot walking, carried out for the inclined surfaces with increased surface friction, along with the frequency-domain analysis of the experimental measurements of the angular-pitch velocity. In Section 5, an extension of the IPM is proposed to incorporate the implemented motor-control system (Section 5.1) and then the IPM is extended to incorporate the nonlinearity due to the surface inclination, in order to explain the higher-order harmonics (Section 5.2) obtained in the measurements. Section 6 concludes the paper.

## 2. Robot-Walking Experiments

### 2.1. Motor Control System

**Figure 4:**
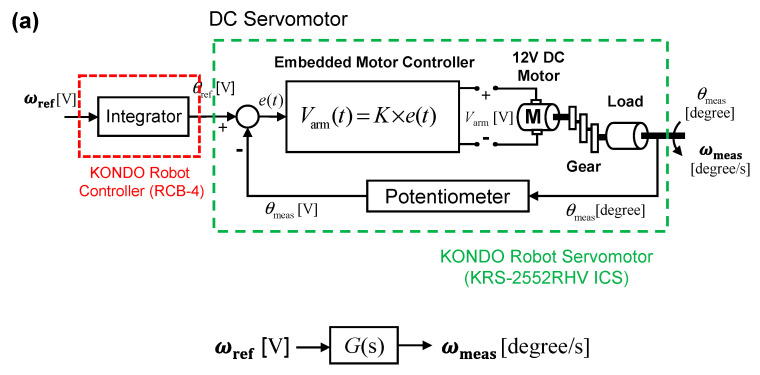

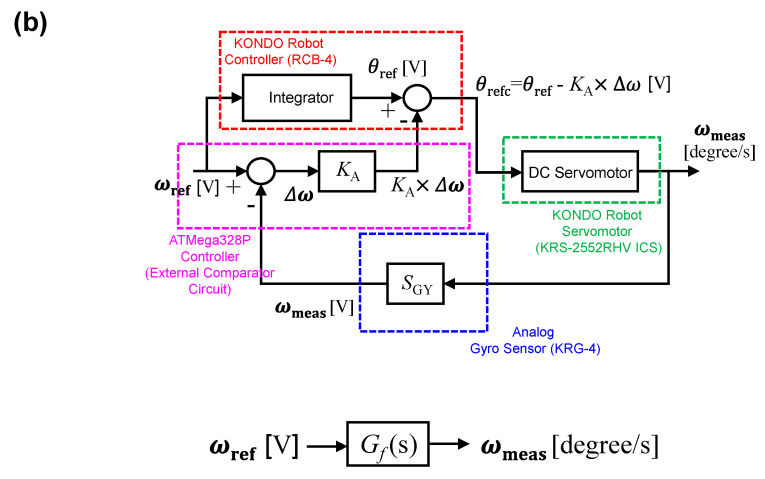
(**a**) Schematic diagram of the internal control circuit of DC servomotor used in the experiment. The transfer-function formulation used for modeling the motor-control system without the gyro-sensor feedback loop. (**b**) Schematic diagram of the motor-control system, implemented for adjusting the robot’s ankle-pitch motor on inclined surfaces. The difference Δ***ω*** between measured angular-pitch velocity and a reference angular-pitch velocity, stored in the robot’s control system, is amplified by factor *K*_A_ for feedback to the ankle-pitch motor.

The robot is equipped with 17 KONDO KRS-2552RHV DC servomotors [49] for walking or performing any other manipulation. Among these 17 motors, four motors, one ankle-pitch motor (M_A_), and one hip-pitch motor (M_H_) on each leg, are controlled in the present investigation, to realize stable walking on the inclined surface. The internal control circuit of each DC servomotor is shown in Figure 4a. The DC servomotor is implemented using a 12V DC geared motor with a potentiometer-based position-feedback loop. The embedded controller, implemented on the PIC16F690 platform [50], controls the DC motor’s armature voltage *V*_arm_, based on the difference between a reference motor angle *θ*_ref_ (in [V]) and the motor angle *θ*_meas_ (in [V]), which is measured by the potentiometer. The reference motor angle *θ*_ref_ (in [V]) is supplied by the robot controller RCB-4 [51] integrating the reference angular-pitch velocity ***ω***_ref_ (in [V]). Please note, that the investigated angular velocity is a one-dimensional vector quantity, which means a positive angular-velocity value refers to the clockwise rotation and a negative angular-velocity value refers to the counter-clockwise rotation. In this way, the DC servomotor transfers the reference angular-pitch velocity ***ω***_ref_ (in [V]) to the angular velocity ***ω***_meas_ (in [degree/s]) of the servomotor. To calculate the output angular velocity, a transfer-function formulation is required, as shown in Figure 4a [52,53,54,55,56,57,58]. The transfer function of the motor-control system without the gyro-sensor-feedback loop can be written in the Laplacian domain (s-domain) (see Figure 4a) as follows:

(1) $$G(s)=\frac{K}{Js^{2}+Bs+K}$$

where *J* and *B* are equivalent inertia elements (inertia and viscous friction of the servo motor) and *K* is the proportional gain of the controller, embedded inside the KONDO servomotor KRS-2552RHV.

The discussed motor-control system is integrated with a gyro-sensor-based feedback controller, as shown in Figure 4b. In the motor-control system with gyro-sensor feedback, the angular-pitch velocity of the robot is measured by a single-axis analog gyro sensor KRG-4 [41], which is attached on the robot torso. The measured angular-pitch velocity (in [V]) by the gyro sensor is given by:

(2) $$\omega_{\mathrm{meas}}\;[V]=S_{\mathrm{GY}}\times \omega_{\mathrm{meas}}\;[\mathrm{degree}/s]$$

where *S*_GY_ is the sensitivity of this single-axis analog gyro sensor, which is found to have a mean value of 0.67 in units of mV∙s/degree. The measured angular-pitch velocity ***ω***_meas_ of the robot (in [V]) is fed back to an external comparator circuit, implemented into an ATmega328P controller [59]. The comparator calculates the difference between the measured angular-pitch velocity ***ω***_meas_ (in [V]) and a reference angular-pitch velocity ***ω***_ref_ (in [V]), which is preprogrammed into the memory of the external controller. This difference is amplified with the externally “tunable” feedback gain *K*_A_ for the ankle-pitch motor (or *K*_H_ for the hip-pitch motor), as shown in Figure 4b. The output of the amplifier is then fed back to the robot-controller RCB-4 [51], to adjust the reference value of the ankle-pitch-motor angle according the following the equation:

(3) $$\begin{array}{l} \theta_{\mathrm{refc}}=\theta_{\mathrm{ref}}-K_{A}\times (\omega_{\mathrm{ref}}-\omega_{\mathrm{meas}})\;[V]\\ =\theta_{\mathrm{ref}}-K_{A}\times \Delta \omega \;[V] \end{array}$$

where *K*_A_ is the tunable feedback gain of the amplifier, *θ*_ref_ is the reference angle for the ankle-pitch motor (in [V]), and *θ*_refc_ (in [V]) is the adjusted reference angle for motor rotation. The adjusted reference angle is then used to control the servomotor rotation, as shown in Figure 4b. For other motors, such as the hip-pitch motors M_H_, the control is independently done in the same way as for motor M_A_.

The feedback gain *K*_A_ (or *K*_H_) increases the sensitivity of the motor-angle control on the comparator output. Therefore, for a higher effect of the comparator output, a larger *K*_A_ value is needed to adjust the motor angle by a larger extent. In the literature of compliance modeling for actuators [60], the gain *K*_A_ affects the stiffness of a motor-load transmission system. The frequency-response analysis of such motor-load-transmission systems shows, that the proportional feedback-gain largely affects the vibrations in the system. Therefore, we have designed a proportional control to analyze the effect of gain *K*_A_ on the system performance, before extending it to controllers having multiple tunable gains, e.g., Proportional-Derivative (PD) controllers. This will become clearer in the following section, when the effect of the gain *K*_A_ for controlling the ankle-pitch motor (and *K*_H_ for hip-pitch motor-control) on the robot’s gait data is discussed in detail.

When the feedback loop with the gyro sensor control is implemented, the viscous friction coefficient is reduced from *B* to *B_f_* as follows:

(4) $$B_{f}=B-K_{A}S_{\mathrm{GY}}$$

where *S*_GY_ is the gyro sensor sensitivity and *K*_A_ is the tunable gain in the feedback loop. Due to the gyro-sensor-feedback loop, the modified transfer function G*_f_* of the overall motor-control system (see Figure 4b) is given by:

(5) $$G_{f}(s)=\frac{(1-sK_{A}S_{\mathrm{GY}})K}{Js^{2}+B_{f}s+K}$$

### 2.2. Experimental Setup

A single-axis gyro sensor is attached on the robot torso together with the robot controller, as indicated in Figure 1. The locations of ankle-pitch motor (M_A_) and hip-pitch motor (M_H_) are also indicated in Figure 1. To analyze the motor-control scheme for robot-posture balancing, a smooth surface is inclined by an angle *φ*, as can be seen in Figure 2. The inclination degree of the surface is gradually increased from 0° in a stepwise manner, until the robot falls down during walking. The angular-pitch velocity of the robot is measured, when the robot walks on this surface. Measurements are performed under the following three conditions:

1. The robot is walking on the inclined surfaces without any gyro-sensor-based feedback control, until it becomes unstable beyond a critical inclination *φ*_cr0_.
2. Beyond the critical inclination *φ*_cr0_, the ankle-pitch motor (M_A_) is controlled by the gyro-sensor feedback. This allows the robot to walk up to a higher critical surface inclination *φ*_cr1_. The robot becomes unstable beyond *φ*_cr1_.
3. The hip-pitch motor (M_H_) is controlled by the gyro-sensor feedback in addition to the ankle motor. This enables the robot to walk stably on inclined surfaces above the critical surface inclination *φ*_cr1_.

This stepwise adjustment of the motor control, based on the gyro-sensor feedback, is schematically illustrated in Figure 2.

### 2.3. Robot-Gait Implementation

**Figure 5:**
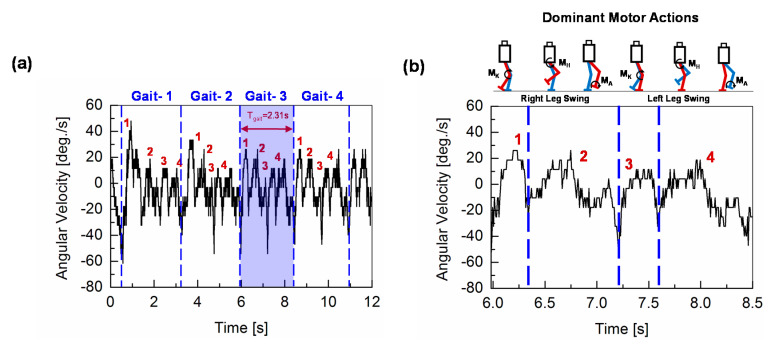
(**a**) Angular-pitch velocity of the robot torso when the robot walks on an uninclined surface (*φ* = 0°). (**b**) The angular-pitch velocity of the third gait cycle as detected by the gyro sensor. The third gait cycle is shown in an expanded form, to explain the gait pattern of the robot on the uninclined surface. Peaks 1 and 2 are due to the positive angular-pitch velocity for the first half of the cycle (right-leg swinging phase), and peaks 3 and 4 are observed in the second half of the cycle (left-leg swinging phase). These four peaks are observed to be the characteristic feature of one gait cycle.

Under a surface inclination of *φ* < *φ*_cr0_, the robot is allowed to walk on the inclined surface without any gyro-sensor-based feedback control of the servomotors. The angular-pitch velocity of the robot is measured, when the robot is walking on an uninclined surface (*φ* = 0°), as schematically illustrated in Figure 3. The gait-cycle period (*T*_gait_) of the robot is set to 2.31 s. One experimental walking period includes a start phase (half a gait), four complete gait cycles, and an end phase (half a gait). The measured angular-pitch velocity ***ω***_meas_ by the gyro sensor in the robot torso for *φ* = 0° is shown in Figure 5a. It can be seen that the pattern of ***ω***_meas_ becomes quite identical after the second complete gait cycle. Therefore, we consider the ***ω***_meas_ waveform of the third gait (Gait-3) cycle for our further analysis of the robot’s walking dynamics. The zoomed waveform of Gait-3 is shown in Figure 5b, where the schematic robot motions of this walking cycle are depicted above the graph. Each gait cycle is characterized by four peaks (1, 2, 3, and 4), as shown in Figure 5b. Peaks 1 and 2 occur due to the positive angular-pitch velocity of the robot while swinging the right leg during the first half of the gait cycle. Similarly, peaks 3 and 4 occur while swinging the left leg during the second half of the gait cycle. These four peaks characterize a gait cycle in the time-domain. The following subsection will discuss how these four peaks change due to the effect of surface inclination and gyro-sensor-based feedback control.

The measured angular-pitch velocity ***ω***_meas_ of the robot torso as a function of time is classified into different phases of the walking cycle. The measured gait cycle for *φ* = 0° is considered as the “fingerprint” data of the robot walking, which provides the basic gait data without specific additional environmental contributions.

### 2.4. Experimental Results

**Figure 6:**
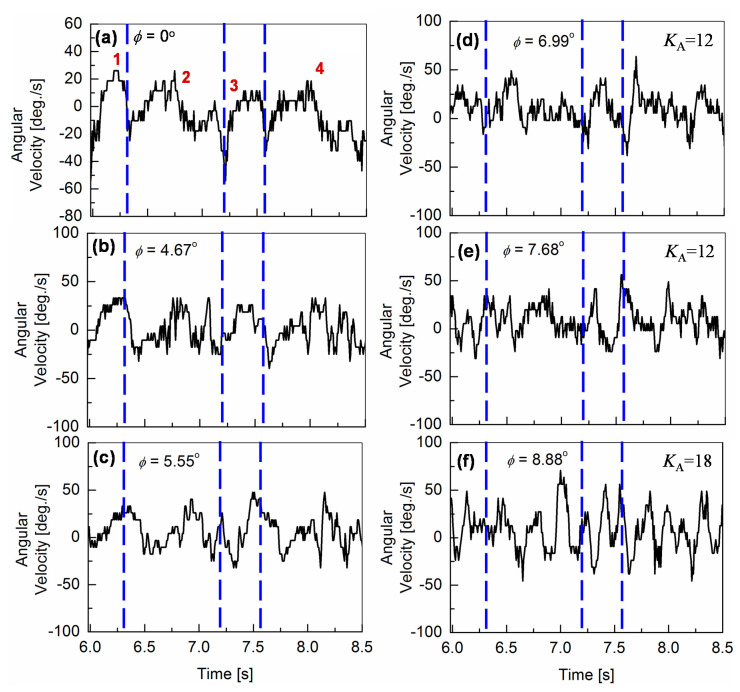
Angular velocities of the robot’s pitch motion during a gait cycle, measured for different surface inclinations. The results are compared with angular-pitch velocities, measured for *φ* = 0° (see (**a**)). An increase in the amplitude and number of oscillations is observed as surface inclination *φ* is increased (see (**b**) and (**c**)). The oscillation increases by greater extent at higher surface inclinations with the feedback control (see (**d**), (**e**) and (**f**)). As the surface inclination is increased, the peaks 1, 2, 3, and 4 of the fundamental gait pattern (for *φ* = 0°) become less distinct due to the increasing additional oscillations.

The angular-pitch velocities ***ω***_meas_ of Gait-3 with different increased surface inclinations *φ* are shown in Figure 6. The robot, walking on an inclined downslope surface without the motor feedback control, is studied first by increasing *φ* from 0° to *φ*_cr0_ = 5.55°. It can be seen, that additional oscillatory patterns appear in the ***ω***_meas_ waveforms, which become more obvious as *φ* increases. The designated peaks 1, 2, 3, and 4 become indistinguishable, due to an amplitude increase in the additional oscillatory patterns. As *φ* approaches *φ*_cr0_, the robot body starts to increasingly vibrate, which makes the robot posture unstable. Beyond the critical surface inclination *φ*_cr0_, the robot falls down when it starts to walk. Therefore, the ankle-pitch motor M_A_ is controlled beyond *φ*_cr0_ of 5.55°. For this control, a proportional control system is applied, which is implemented into the robot, as shown in Figure 4a. This proportional control system uses an amplifier gain *K*_A_ to control the motor angle, where *K*_A_ = 12 makes the robot walking again stable beyond *φ*_cr0_, as shown in Figure 6. At lower values of *K*_A_, the torque at the ankle-pitch motor is not sufficient to stop the robot from falling down. With *K*_A_ = 12, the robot can walk on surfaces with inclinations up to *φ* = 8.88°. Beyond *φ*_cr0_ = 5.55°, it is observed that the oscillatory nature of the angular-pitch velocity increases largely due to the implementation of the gyro-sensor-based feedback-control system. The characterizing peaks (1, 2, 3, and 4) are no more observed distinctly and the pattern of the third gait cycle becomes more oscillatory than the fundamental gait data (Figure 6a). At *φ* = 8.88°, it is observed, that even though the robot can walk on the downslope, it starts slipping on the surface. If *K*_A_ is increased further to 18, the slipping is reduced and the robot can walk down the slope more safely. However, the robot body vibrates at a much higher frequency, than observed for a surface inclination of *φ* = 5.55°.

**Figure 7:**
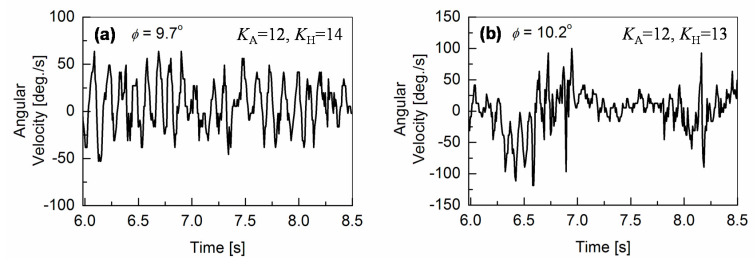
Angular-pitch velocity for a gait cycle with both ankle-pitch-motor control and hip-pitch-motor control, measured at different higher surface inclinations. (**a**) Robot vibrations increase for *K*_H_ = 14 in the hip-pitch-motor control. (**b**) *K*_A_ = 12 and *K*_H_ = 13 are an optimized motor-control setting, allowing stable down-slope walking up to inclinations of *φ* = 10.2°.

At *φ* = 9.7°, the robot starts to slip again, even with *K*_A_ = 18. If we increase *K*_A_ to more than 18, the vibrations of the robot body dominate the robot motion, which makes the robot very unstable during walking. Therefore, the hip-pitch motor M_H_ is additionally controlled from *φ* = 9.7°, in the same way as M_A_ but independently. This M_H_ motor control, together with keeping the *K*_A_ value in the M_A_ control at 12, reduces again the vibrations. The feedback gain *K*_H_ for the hip-pitch-motor control is varied from 12 to 14. It is observed, that the vibration increases at the higher value of *K*_H_ = 14 (see Figure 7a), whereas the slipping dominates at lower values such as *K*_H_ = 12, similar to the results for the ankle-pitch-motor control with *K*_A_. Therefore, we set the *K*_H_ to 13, where both slipping and vibrations are optimized, and the robot can walk down the slope without falling up to inclinations of *φ* = 10.2°, as verified in Figure 7b. For *φ* > 10.2°, the robot walking becomes again unstable, mostly due to an increased slipping.

## 3. Analysis of Experimental Results

**Figure 8:**
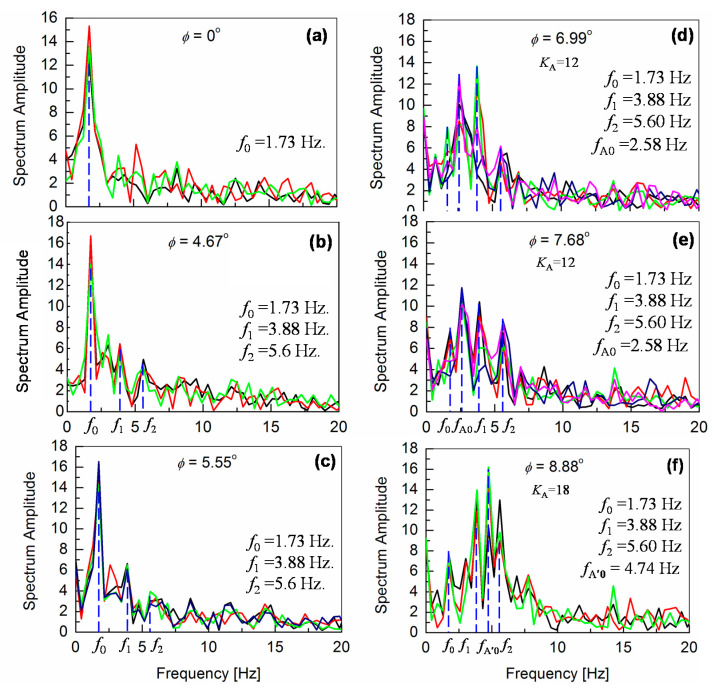
Fourier analysis of the angular-pitch velocity waveforms measured by the gyro sensor for the robot-body, when the robot is walking stably on inclined surfaces without ((**a**), (**b**), (**c**)) and with ((**d**), (**e**), (**f**)) gyro-sensor feedback. The frequency *f*_A0_ = 2.58 Hz (see (**d**) and (**e**)) is induced by the motor feedback with *K*_A_ = 12, which is shifted to *f*_A’0_ = 4.74 Hz (see (**f**)) due to an increased *K*_A_. Further explanations are given in Section 5.1.

**Figure 9:**
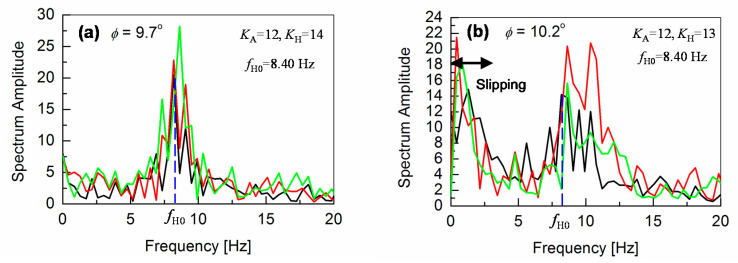
Fourier transformation of the angular-pitch velocity, when both ankle-pitch and hip-pitch motors are controlled by the gyro sensor for *φ* = 9.7° (see (**a**)) and *φ* = 10.2° (see (**b**)). It can be seen that the contribution due to the fundamental walking frequency of 1.73 Hz is no more observable. Instead, a peak at higher frequency is observed. The measured time-domain waveform becomes more oscillatory, as shown in Figure 7.

The experimental results revealed, that the motor-feedback control enables the robot walking on inclined surfaces, but induces vibrations of the robot-body at the same time. Here, our focus is given on the origin of the vibrations. It can be observed in Figure 6 and Figure 7, that the ***ω***_meas_ waveform becomes complex and that no clear gait-pattern characteristics is visible at higher surface inclinations. To investigate the origin of the oscillation increase, a Fourier transformation is performed for the time-domain measurements, when the robot is still walking on inclined surfaces rather stably. Figure 8 and Figure 9 depict the results for measured angular-pitch velocity waveforms ***ω***_meas_ in the frequency domain. Without the M_A_ control for *φ* ≤ 5.55°, a single dominant peak at *f*_0_ = 1.73 Hz is observed, which refers to the fundamental walking frequency (see Figure 8b,c). Additional noise-like peaks could be due to higher-order harmonics, which originate from the nonlinear motor torque. The same measurements are repeated several times, to verify the statistical significance, and are plotted together in the graphs of Figure 8.

For *φ* **≥** 6.99°, the feedback mechanism is implemented with different *K*_A_ values, as shown in Figure 8d–f. It is observed that contributions up to the second-order harmonics (*f*_1_ and *f*_2_) are significant. These higher frequency oscillations refer to the oscillation increase in the time-domain measurements. Here, an additional frequency peak *f*_A0_ is observed at 2.58 Hz, as seen in Figure 8d,e, and originates from the gyro-sensor-based feedback control of the ankle-pitch motor.

As the surface inclination increases further above *φ* = 8.88°, oscillation frequencies are shifted to higher values. This shift to higher frequencies becomes quite drastic when introducing the *K*_H_ control, as can be seen in Figure 9. Under such a condition, slipping of the robot feet becomes quite clear, and the robot-gait pattern is no more detectable in the time domain measurements. Since our measurements are performed with slippery robot feet on a smooth surface, to investigate the robot-balancing mechanism, the measurements are valid for inclinations before the robot slipping dominates. This inclination limit is *φ* < *φ*_cr1_ = 8.88°. At the surface inclination of *φ =* 8.88°, an increased feedback gain *K*_A_ of 18 is required, which causes a larger oscillatory behavior of the robot, as shown in Figure 6f.

**Figure 10:**
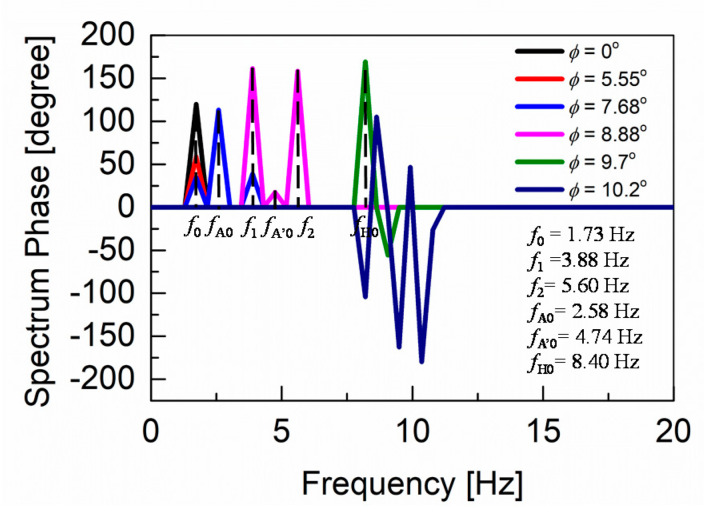
Phase plots for the measured pitch velocity in the cases of various surface inclinations. The contributing frequencies are indicated, confirming the findings from the corresponding amplitude plots. A tolerance value of 33% of the absolute maximum in the amplitude spectrum is used for the phase-plot calculation.

**Figure 11:**
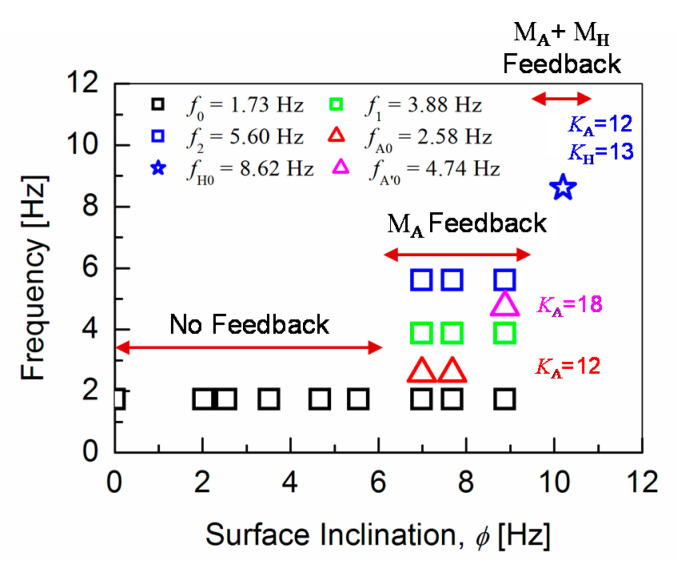
Contributing frequencies as extracted from the frequency-response analysis of the angular-pitch velocity as a function of surface inclination *φ*.

Frequency responses of the angular-pitch velocity at *φ* = 9.7° and *φ* = 10.2° are shown in Figure 9a,b, respectively. The frequency peaks are observed at higher frequencies between 7.5 and 10 Hz for both cases. The corresponding phase plots (spectrum phase vs. frequency) of the angular-pitch velocity are shown in Figure 10. A relatively high tolerance value of 33% of the maximum absolute value in the amplitude spectrum is used during the calculation of these phase plots, to remove the unwanted effects of the quite large noise in the measured time-domain data. The phase plots show the expected peaks at the contributing frequencies, as supported by the amplitude plots for the angular-pitch velocity. The data suggests, that the linearity of the gait pattern is completely lost, as can be observed in particular from the diminished amplitude at *f*_0_ = 1.73 Hz. Figure 11 summarizes the observed dominant frequency peaks as a function of the surface inclination *φ*. Basically, the regular gait pattern of *f*_0_ and its harmonics are observed. By introducing the feedback control, however, additional control frequencies appear. These frequencies must be kept at higher than *f*_0_, to sustain the robot-body posture while walking and are observed as strong robot-body vibrations. By increasing the *K*_A_ value to 18, the additional frequency due to the feedback increases up to *f*_A’0_ = 4.74 Hz, which dominates the walking condition at the same time (see Figure 8f). The introduction the *K*_H_ control causes an additional frequency at *f*_H0_ = 8.40 Hz, as shown in Figure 9a. This relatively high frequency unfortunately controls the gait pattern of the robot, making the robot unstable due to increased vibrations. By increasing the surface inclination up to *φ* =10.2°, strong peaks appear also at a low frequency (see Figure 9b), which are attributed to the slipping of the robot’s feet.

## 4. Experiments with Robot-Foot Friction

**Figure 12:**
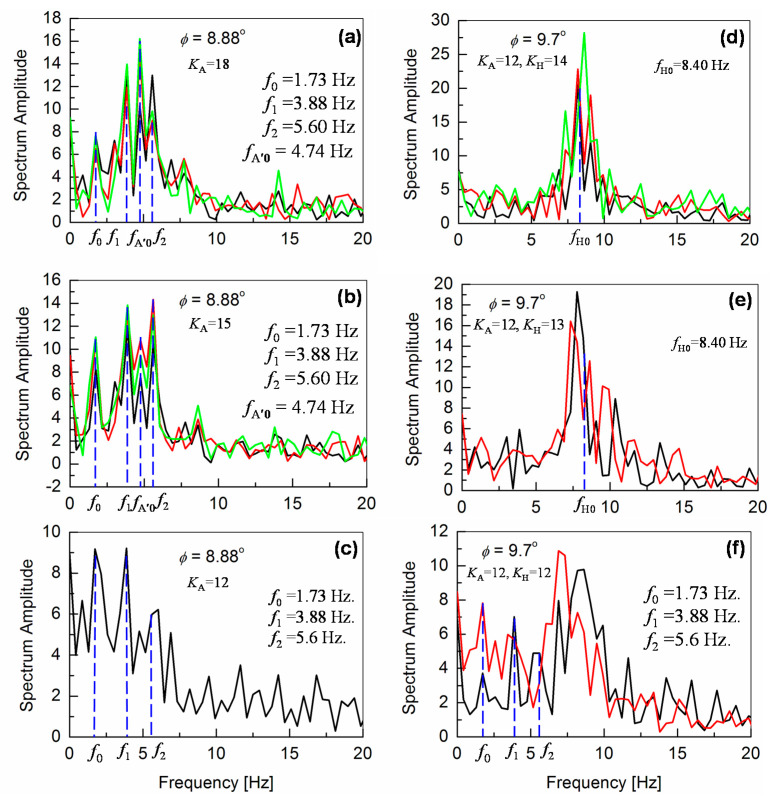
Fourier analysis of the angular-pitch velocity of the robot walking on an inclined surface for *φ* = 8.88° with varying *K*_A_ (18 in (**a**), 15 in (**b**), and 12 in (**c**)) and for *φ* = 9.7° with varying *K*_H_ (14 in (**d**), 13 in (**e**), and 12 in (**f**)). By reducing *K*_A_ and *K*_H_ values, the robot posture becomes unstable due to slipping, as shown in (**b**) and (**c**).

To improve the walking stability, the ankle-pitch motor angle *θ*_A_ as well as the hip-pitch motor angle *θ*_H_ are controlled independently with the information obtained by the gyro sensor. Though it was found that the increase of the feedback control reduces slipping of the robot feet, the robot-body vibrations increase with increasing the feedback gains *K*_A_ and *K*_H_. Therefore, it is concluded that the enhanced feedback control of the presented implementation is an insufficient solution for maintaining the stable robot posture. The reason for the robot instability on an inclined surface is due to the additional force component induced by the slope, which causes a nonlinearity of the motor torque. This nonlinearity introduces the higher-order harmonics (*f*_1_ and *f*_2_) of the gait pattern. Another important observation is that the robot-body vibrations cause an unstable robot posture. These robot vibrations can be attributed to the posture-angle calibration through the motor-feedback control. Figure 12a–c summarizes the features of the *K*_A_ control for *φ* = 8.88°. Even though the robot feet become quite slippery, the fundamental walking frequency *f*_0_ is clearly observed for all *K*_A_ values. With the increased *K*_A_ value, it can be seen that the nonlinearity of the gait pattern increases. The nonlinearity continues to increase with the additional control of *K*_H_. This can be seen in Figure 12d–f for *φ* = 9.7°, where the newly appearing higher frequencies between 7.5 and 10 Hz are due to the feedback-motor-control system for the hip-pitch motor M_H_.

**Figure 13:**
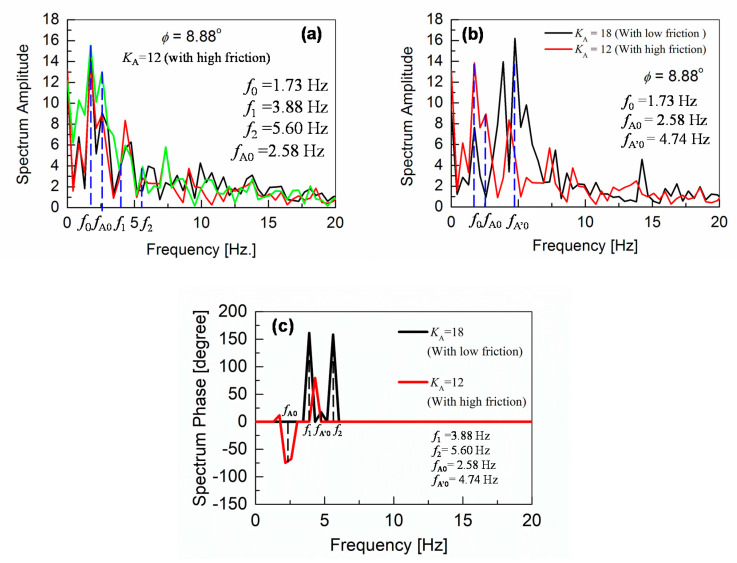
(**a**) Amplitude plot, which shows the Fourier analysis of the angular-pitch velocity data when the robot is allowed to walk with higher feet friction and reduced value of *K*_A_ = 12 on a surface of *φ* = 8.88° inclination. (**b**) Comparison of amplitude plots, which demonstrates the shift in the additional frequency due to the feedback control from *f*_A’0_ = 4.74 to *f*_A0_ = 2.58 Hz, when lowering *K*_A_ from 18 to 12, as possible by the increased feet friction. (**c**) Phase plot, which confirms the frequency-peak shift to a lower value, due to lowering of the feedback gain *K*_A_ at a higher surface friction. A tolerance value of 33% of the absolute maximum in the amplitude spectrum is used for the phase-plot calculation.

Up to now, our experiments have been carried out for the robot walking on inclined surfaces with low friction, to verify major factors which prevent the robot from stable walking. In this section, further experiments with an increased surface friction are described, where rubber soles are attached to the robot feet. The experiments are performed at *φ* = 8.88°. The results with *K*_A_ = 12 are shown in Figure 13a. With low friction (see Figure 12a), the frequency *f*_A0_ = 2.58 Hz is not observable, but shifted to *f*_A’0_ = 4.74 Hz due to the increased value of *K*_A_ = 18 to make the robot-posture stable. Though the robot was able to walk on the surface rather stably only with *K*_A_ = 18 for the low friction surface, the robot could walk stably even with *K*_A_ = 12 in the case of increased friction. In Figure 13b, the two results for different friction conditions are compared. The frequency *f*_0_ becomes dominant and the *f*_A0_ reappears for the high friction case. The spectrum-amplitude plot is supported by the corresponding phase plot in Figure 13c, which shows shifting of the frequency peaks to lower values, due to lowering of the feedback gain *K*_A_ at a higher surface friction.

**Figure 14:**
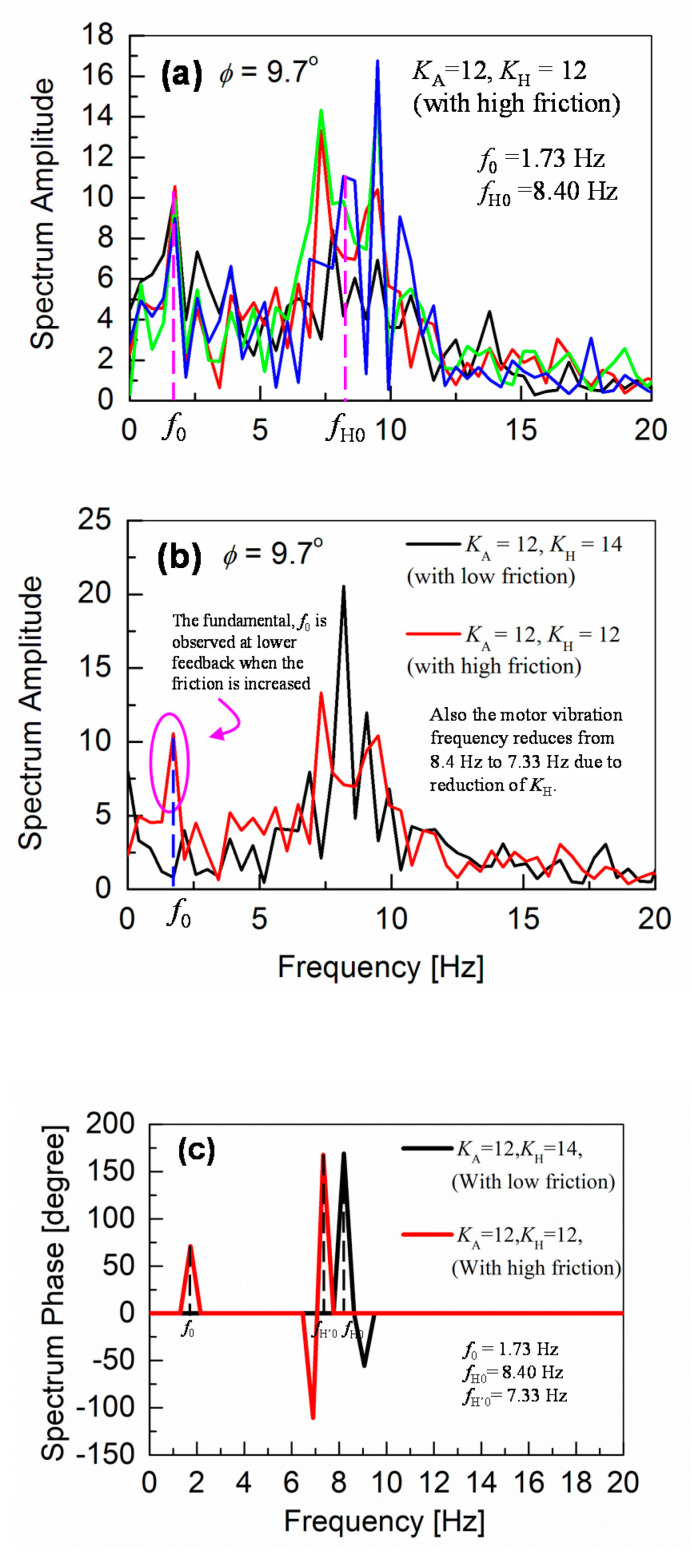
(**a**) Shows that robots can walk on a surface of *φ* = 9.7° inclination with *K*_H_ =12, when the robot-feet friction is increased. (**b**) Illustrates two important features, which result from the increased-friction-enabled *K*_H_ lowering: First, lowering of the frequency, induced by the additional hip-pitch motor control, from 8.40 to 7.33 Hz. Second, reappearance of the peak at a fundamental walking frequency *f*_0_ = 1.73 Hz. (**c**) The corresponding phase plot, which supports the appearance of the peak at *f*_0_ and the lowering of *K*_H_. A tolerance value of 33% of the absolute maximum in the amplitude spectrum is used for the phase-plot calculation.

Similar measurement results for *φ* = 9.7°, with increased friction, are shown in Figure 14a. The comparison with the low-friction results, where the robot needed a higher *K*_H_ value of 14 for stable walking, is given in Figure 14b. Two important improvements in the gait-pattern are clearly observed. First, we were successful to achieve, a stable walking with a lower *K*_H_ value of 12, having a peak at a lower frequency of 7.33 Hz (compared to 8.40 Hz at *K*_H_ = 14 for the lower friction case). Second, the reappearance of the fundamental walking frequency *f*_0_ is confirmed, as highlighted by the encircling in the graph, which is also supported by calculating the phase plot of the angular-pitch velocity, as shown in Figure 14c. This phase plot further verifies, that a frequency peak appears at *f*_0_ in addition to the lowering of the contributing frequency from 8.40 to 7.33 Hz, thus supporting the results obtained from the spectrum amplitude plot (see Figure 14b). Therefore, the high-frequency vibration, observed around 8.40 Hz, is drastically reduced in magnitude and frequency, leading to a more stable gait pattern of the robot.

## 5. Model Development for Robot Balancing

### 5.1. Inverted Pendulum Model for Robot Walking

**Figure 15:**
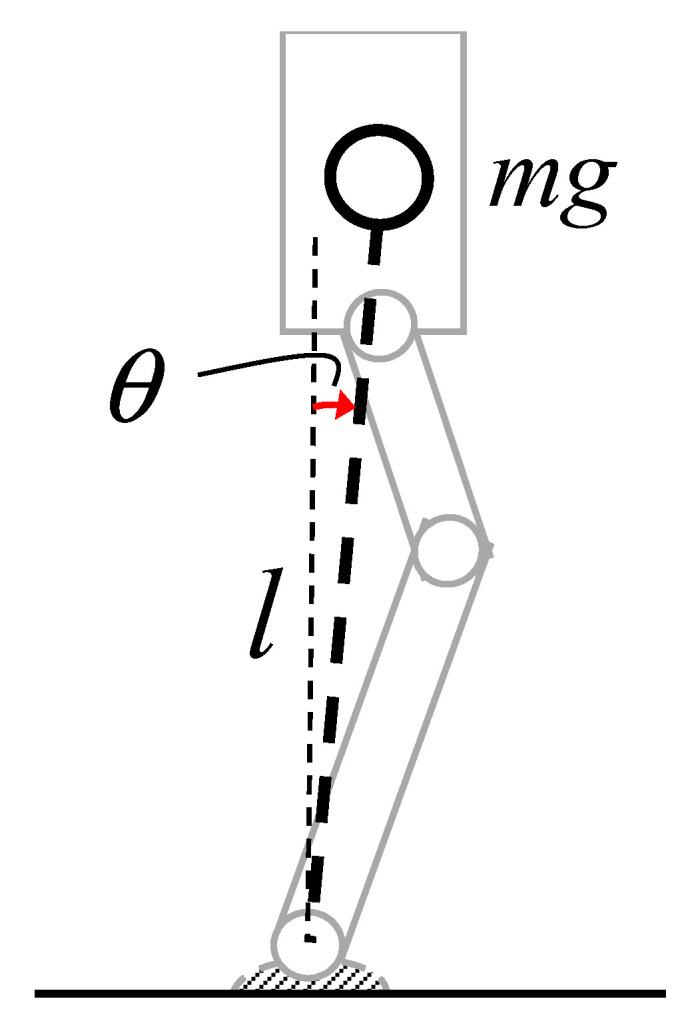
The inverted pendulum model (IPM) to explain the robot walking dynamics. The parameters *θ*, *m*, *l,* and *g* are the angle of robot-torso rotation in the sagittal plane, robot mass, robot-center-of-mass distance from the ankle-pitch motor, and acceleration due to gravity, respectively.

For modeling the walking dynamics of the humanoid robot, a basic inverted pendulum in polar coordinates is considered, as shown in Figure 15, which can be written as [15,61].

(6) $$ml^{2}\frac{d^{2}\theta (t)}{dt^{2}}-mgl\sin\theta (t)=0$$

where *θ*, *m*, *l,* and *g* are the angle of pitch rotation of the robot torso in the sagittal plane, robot mass, distance of robot’s COM from the ankle-pitch motor, and acceleration due to gravity, respectively, as also indicated in Figure 15. The physical parameters of the robot are given in Table 1. The first derivative of θ(*t*) with respect to time is the angular-pitch velocity, measured in our experiments (see Figure 6 and Figure 7). Under the condition of small *θ*(*t*), the solution of Equation (6) becomes sinusoidal and the angular-pitch velocity can be written as follows:

(7) $$\omega (t)=a\cos2\pi f_{n}t$$

Let *a* and *f*_n_ be the initial angular-pitch velocity and the natural frequency of the inverted pendulum, respectively, where *f*_n_ is given by [61]:

(8) $$f_{n}=\frac{1}{2\pi }\sqrt{\frac{g}{l}}=0.9\;\mathrm{Hz}$$

Therefore, the ideal walking motion of a humanoid robot can be modeled as the repetitive (or periodic) motion of the inverted pendulum. In our investigation, one gait cycle can be divided into two half-cycles, where one half cycle is due to the left-leg movement and the other is due to the right-leg movement. Now, in each half cycle there are two motion phases: One Single Support Phase (SSP) and one Double Support Phase (DSP). The SSP of the left and the right leg can be treated identically. This is quite reasonable, since only frequency *f*_0_ = 1.73 Hz is observed in the gait pattern. During the DSP motion, only the active-leg exchange happens, and thus the gait pattern is characterized nearly exclusively by SSP in the frequency-domain analysis. Therefore, based on the experimental observation and the outlined theoretical justification, we can conclude that the fundamental walking frequency *f*_0_ of the robot’s gait pattern should be twice of the natural frequency *f*_n_ of the IPM, given by Equation (8). The theoretically calculated *f*_0_ of 1.8 Hz is indeed close to the experimentally extracted value of 1.73 Hz.

Under an ideal walking condition, the reference angular-pitch velocity ***ω***_ref_ is thus given by a single frequency *f*_0_ with amplitude *a*:

(9) $$\omega_{\mathrm{ref}}(t)=a\cos2\pi f_{0}t$$

Consequently, only one frequency peak should be observed at the *f*_0_ = 1.73 Hz in the Fourier transformation output for *φ* = 0, i.e., for an uninclined surface. This means, there is no oscillatory nature induced in the output other than the input frequency.

The output of the modified control system with gyro-sensor feedback (see Figure 4b), in response to a sinusoidal input (Equation (9)) of frequency *f*_0_ = 1.73 Hz, is then given by:

(10) $$\omega_{0}(s)=\frac{(1-sK_{A}S_{\mathrm{GY}})K}{Js^{2}+B_{f}s+K}\times \frac{s}{s^{2}+{\left(2\pi f_{0}\right)}^{2}}$$

where the transfer function of the system is given by Equation (5). In the time domain, the output angular-pitch velocity can be obtained by an Inverse-Laplace Transform [52] of Equation (10) as:

(11) $$\omega_{0}(t)=a_{0}\cos(2\pi f_{0}t)+b_{0}e^{-\;\frac{B_{f}}{J}t}\cos(2\pi f_{A0}t)$$

where the frequency *f*_A0_ is the damped natural frequency of the motor-control system for the ankle-pitch motor. The two amplitudes *a*_0_ and *b*_0_ can be written as [61]:

(12) $$f_{A0}=\sqrt{\frac{K}{J}\left(1-\frac{B_{f}{}^{2}}{JK}\right)}=\sqrt{\frac{K}{J}\left(1-\frac{{\left(B-K_{A}S_{\mathrm{GY}}\right)}^{2}}{JK}\right)}$$

(13) $$a_{0}=\frac{a}{\sqrt{{\left(K-J{\left(2\pi f_{0}\right)}^{2}\right)}^{2}+{\left(2(B-K_{A}S_{\mathrm{GY}})\pi f_{0}\right)}^{2}}}$$

(14) $$b_{0}=\sqrt{\;{\left[\frac{B_{f}}{4\pi Jf_{0}}{\omega_{0}(t)|}_{t=0}+\frac{1}{2\pi f_{0}}{\frac{d\omega_{0}(t)}{dt}|}_{t=0}\right]}^{2}+{\omega_{0}(t)|}_{t=0}}$$

Therefore, the measured output for the angular-pitch velocity ***ω*_0_**(*t*) should show fundamental frequency peaks at two different frequencies *f*_0_ and *f*_A0_ in the corresponding Fourier transform. From Equation (12), it can be seen, that as the feedback gain *K*_A_ is increased, the frequency *f*_A0_ also increases. This is confirmed by the experimental observations in Figure 8d–f.

### 5.2. Robot-Walking-Model Extension to Inclined Surfaces

The analysis, carried out in the last subsection, assumed the dynamic system to be linear, which allows the use of transfer-function modeling. The time-domain output (Equation (11)) is obtained to explain the experimentally-measured results. The model (Equation (11)), discussed in the last subsection, explains the output of a second-order system (Equation (5)) in response to a sinusoidal input of frequency 1.73 Hz. The output consists of the input frequency of 1.73 Hz and the damped natural frequency *f*_A0_.

Here, the model (see Equation (10)) is extended to include the nonlinearity of the robot dynamics, which is induced in the inverted pendulum by the surface inclination *φ*. First, the origin of higher-order harmonics is explained and then the inclusion of the harmonics (nonlinearity) into the model (Equation (11)) is discussed. For understanding the nonlinearity, the time-domain analysis of a nonlinear pendulum is undertaken. For an inclined surface, Equation (6) changes to [15,62]:

(15) $$ml^{2}\frac{d^{2}\theta (t)}{dt^{2}}+mgl\sin(\theta (t)+\phi )=0$$

where *φ* is the surface inclination. If we expand the sinusoidal term following the basic trigonometric identity [63], we get the following:

(16) $$ml^{2}\frac{d^{2}\theta (t)}{dt^{2}}+mgl\left(\cos\phi \;\sin\theta (t)+\sin\phi \;\cos\theta (t)\right)=0$$

This result can be rewritten by series expansions of sin*θ*(*t*) and cos*θ*(*t*) into the following form:

(17) $$ml^{2}\frac{d^{2}\theta (t)}{dt^{2}}+mgl\cos\phi \;\left(\theta (t)-\frac{\theta^{3}(t)}{6}+\frac{\theta^{5}(t)}{120}-\ldots \right)+mgl\sin\phi \;\left(1-\frac{\theta^{2}(t)}{2}+\frac{\theta^{4}(t)}{24}-\ldots \right)=0$$

The solution of Equation (17) with a sinusoidal input of the fundamental walking frequency *f*_0_ (see Equation (9)) of the robot’s gait pattern is given by [64,65,66,67,68,69,70,71,72]:

(18) $$\omega_{\mathrm{meas}}(t)=A_{0}+a_{0}\cos(2\pi f_{0}t)+a_{1}\cos(2\pi f_{1}t)+a_{2}\cos(6\pi f_{2}t)$$

where *a*_0_ is the amplitude of the fundamental walking-frequency component, given by Equation (13). The amplitudes *a*_1_ and *a*_2_ of the harmonics are functions of the surface inclination *φ*, as shown in Equations (19) and (20):

(19) $$a_{1}=\frac{g\cos\phi }{6l}a_{0}$$

(20) $$a_{2}=\frac{g\sin\phi }{2l}a_{0}$$

In both time-domain (Figure 6 and Figure 7) and frequency-domain (Figure 8 and Figure 9), it is experimentally observed that the oscillation amplitudes become larger with an increase in *φ*. From the above investigation, our experimental findings are therefore theoretically confirmed. The surface inclination *φ* has two main effects on the measured angular-pitch velocity of the robot. First, there is an increase in nonlinearity, which causes the appearance of higher-order harmonics. Second, the oscillation amplitudes increase for a larger surface inclination. From the results, it can be confirmed, that the harmonic coefficients *a*_1_ and *a*_2_ give dominating contributions of the robot motion under high *φ* values. Equation (18) explains the trend of the measured angular-pitch velocity for surface inclinations *φ* ≤ 5.55° (see Figure 8b,c)) and the nonlinearity origin, responsible for the presence of the higher-order harmonics of the fundamental frequency at 1.73 Hz. It is now important to incorporate these higher-order harmonics into the previously discussed model (Equation (11)).

If the feedback control of *K*_A_ is applied for *φ* ≥ 5.55°, an additional frequency peak is observed (see Figure 8d–f). The origin of the peak strength at this additional frequency *f*_A0_ is explained by a low damping, as apparent in Equation (11). The theoretical expression for *f*_A0_ is given by Equation (12).

The experimentally measured results can be explained by calculating the output of the second-order system (Equation (5)) in response to an algebraic sum of sinusoids with the fundamental frequency and its higher harmonics.

(21) $$\omega_{\mathrm{meas}}(s)=\frac{(1-sK_{A}S_{\mathrm{GY}})K}{Js^{2}+B_{f}s+K}\times \left(\frac{s}{s^{2}+{\left(2\pi f_{0}\right)}^{2}}+\frac{s}{s^{2}+{\left(2\pi f_{1}\right)}^{2}}+\frac{s}{s^{2}+{\left(2\pi f_{2}\right)}^{2}}\right)$$

The measured angular-pitch velocity can be analyzed with the following equation, obtained by the inverse-Laplace transform of Equation (20).

(22) $$\omega_{\mathrm{meas}}(t)=A_{0}+\sum_{n=0}^{2}a_{n}\cos2\pi f_{n}t+B_{0}+b_{0}e^{-\;\frac{B_{f}}{J}t}\cos2\pi f_{A0}t$$

where ***ω***_meas_ (*t*) is the measured angular-pitch velocity when the gyro-sensor feedback is applied for the motor control. As the surface inclination *φ* is increased, the feedback strength *K*_A_ has to be also increased to keep the robot posture stable. However, with increasing *K*_A_ the vibration of the robot body also increases. The frequency-domain analysis of the angular-pitch velocity is dominated by the *K*_A_-induced damped natural frequency *f*_A0_, given in Equation (12), and the gravity-induced harmonics of the fundamental walking frequency *f*_0_, given in Equation (18). Nevertheless, to keep a sufficiently stable robot walking, the amplitude of *f*_0_ must dominate over the other frequencies.

At higher inclinations of 9.7° and 10.2°, the *f*_0_ contribution is no more observable, as evident in Figure 9. Therefore, a sufficiently stable robot walking cannot be achieved anymore, due to the large vibrations at higher frequencies, meaning that the fundamental gait pattern of the robot is no more present. Therefore, one way to maintain a stable gait pattern is to minimize the addition of higher frequencies, other than *f*_0_ and its harmonics (2*f*_0_ and 3*f*_0_). The other way to minimize the high-frequency vibrations is the strength adjustment of the feedback gains (*K*_A_ and *K*_H_), which must be kept low enough, so that the robot’s fundamental walking frequency *f*_0_ still remains observable.

## 6. Conclusions

We have investigated the origin of the robot-posture instability, which occurs during walking on inclined surfaces. It was found, that the higher-order harmonics are induced by the nonlinearities of the motor rotations, caused by the distorting effects of the gravity force. The contribution of the harmonics increases when the surface inclination increases, resulting in the robot falling down. To protect the robot from falling down, a motor-feedback control has been implemented in the robot system. Here, it was found, that the feedback-control contribution must be kept smaller than the normal walking contribution, so that a clear fundamental walking frequency is still observable. However, it can easily happen, that the necessary feedback control becomes too large for maintaining the fundamental robot posture on a largely inclined surface. Therefore, a strongly enhanced vibration of the robot body is caused, which becomes an important origin of the instabilities under a large surface inclination. For solving this stability problem, it was further demonstrated, that a larger friction between the robot feet and the surface provides an efficient improvement to keep the feedback-induced oscillations at higher frequencies sufficiently small. In summary, the frequency-domain analysis provides a powerful tool for understanding the gait pattern of the robot and for deriving suitable methods of gait-pattern stabilization under external disturbances.

## Figures and Tables

**Table 1:** KONDO KHR-3HV robot physical parameters.

Parameter	Unit	Amount
Height	m	0.4
Height of COM	m	0.3
Length of foot	m	0.03
Mass ^+^	kg	1.5
Degrees of Freedom *	-	17

^+^ Robot mass is measured after integrating all sensors and the controller in the backpack. * The degree of freedom for the robot is given by the number of motors used in the robot. However, for walking analysis and balancing, we considered just the four motors M_A_ (ankle-pitch motor) and M_H_ (hip-pitch motor) in each leg. These motors are shown in the Figure 1 and Figure 2.
